# Consumers' decoy effect when purchasing pork with traceability technologies

**DOI:** 10.3389/fpubh.2022.941936

**Published:** 2022-07-29

**Authors:** Mo Chen, Pingping Liu, Linhai Wu

**Affiliations:** ^1^School of Economics and Management, Nanjing University of Aeronautics and Astronautics, Nanjing, China; ^2^School of Business, Institute for Food Safety Risk Management, Jiangnan University, Wuxi, China

**Keywords:** traceable pork, decoy effect, individual characteristics, negative binomial count regression, food safety

## Abstract

Despite government investment, policy guidance, and publicity, it has been difficult to establish a traceable food market in China over the past 2 decades. Once a food safety problem occurs, it is difficult to implement effective traceability, recall, and accountability along the food supply chain. How to use the decoy effect to promote the development of China traceable food market? As bounded rationality, a decoy effect exists when adding an alternative to a choice set increases the chance an existing alternative to be chosen. However, few studies have examined the decoy effect in food purchases. Based on consumers in Wuxi, Jiangsu Province, China, we show the decoy effect in traceable pork hindquarter purchases and that the effects differ across product quality and price attributes. The effects are heterogeneous across consumers and are less likely to occur among those who had a personal annual income of more than 50,000 yuan (USD $7,000), were married, and had minor children in the family. These findings have implications on leveraging the influence of the decoy effect on consumer behavior and facilitating the construction of food traceability systems.

## Introduction

Studies on consumer preferences are often based on the assumption of rational behavior, that is, consumer preferences that satisfy completeness, transitivity, and independence of irrelevant alternatives (1). However, many studies have shown that consumer behavior does not always satisfy all the three characteristics. For example, a decoy effect that violates the independence of irrelevant alternatives is commonly found in consumer preferences, as initially defined by Heath and Chatterjee (2). A decoy effect occurs when the addition of a decoy product or product profile to a core set of products makes the target product or target product profile in the core set more attractive and thus more likely to be chosen by a consumer (3, 4). Gonzalez et al. (5) suggested that the addition of an asymmetrically dominant decoy product or product profile shifts consumer preferences to favor the target product or product profile, indicating bounded rationality. This suggests that the decoy effect results from bounded rational consumption. Consumer behavior and the decoy effect are further associated, whereby the more easily a consumer group is decoyed by a decoy product, the stronger the decoy effect may be on their consumption.

A number of studies have examined the decoy effect. Lin et al. (6) found that limited decision time increased the decoy effect, that is, consumers having insufficient time to evaluate the utility of each product make choices by simply comparing the products on the most salient attribute. Malkoc et al. (7) further argued that negative attributes of a product can reduce the decoy effect because consumers demonstrate low attention to negative attributes. Similarly, Malkoc et al. (8) believed that when consumers make decisions about disliked product options, the decoy effect is weakened due to low product utility and psychological resistance. Frederick et al. (9) found that the use of perceptual stimuli can impair consumer comparison of product utility, thus reducing the decoy effect.

Although the decoy effect and the background under which it occurs have been shown by many studies on household consumer products [e.g., (10)], little is known about those on food products. At the same time, the traceable food market has not really been established effectively in China in terms of the current situation in China. Chinese domestic academic circles have also carried out some research on this issue, but those are mainly based on the rational consumption behavior of consumers and seldom have focused on how to use the decoy effect to promote the development of China traceable food market from the perspective of irrational consumption. In the current study, we investigated the decoy effect in food purchase behavior in the case of traceable pork hindquarters and determined the relationship between individual characteristics and the decoy effect in China, which should provide a theoretical basis for promoting traceable food in China and providing consumers with more traceability information. However, although food has the general attributes of ordinary commodities, such as use value, it also has special attributes that differ from those of ordinary commodities because food safety is closely related to individual and public health (11). Therefore, this study does not encourage the abuse of the decoy effect in food purchase, especially its use to market foods that do not comply with laws and regulations. The primary purpose of studying the decoy effect in food market behavior is to protect the normal operation of the food market under relevant laws and to protect the legitimate rights and interests of consumers in the consumption of food, which is closely related to personal health and welfare.

It should be pointed out that there are also other types of irrational behaviors of consumers, such as compromise and anchoring effects, which are sometimes confused. The compromise effect states that a consumer is more likely to choose the middle or compromise option of a choice set, rather than the extremes, thus leading to a larger share of that option in the choice set. The compromise effect is most likely to occur in the choice decision-making process of consumers (12). Anchoring is a bias in which judgments, estimates, or decisions made by consumers in uncertain situations are affected by the initial reference information (initial anchor), making their subsequent estimates biased toward the initial anchor (13). It can be seen that the decoy, compromise, and anchoring effects can be easily distinguished by comparing their concepts.

## Literature review and hypotheses

It is generally believed that the decoy effect is caused by two main factors, namely, decision simplification and utility evaluation of product attributes. (1) Decision simplification: According to Ratneshwar et al. (14), consumers may have difficulty comparing various options in a core set of products if they are unfamiliar with the products and their attributes. The introduction of decoy products can facilitate the pairwise comparison between products (15). The decoy products may highlight the relative advantages and disadvantages of various options in the core set of products, thereby reducing the search costs for product information, simplifying product attribute trade-offs, and altering consumer purchase decisions. (2) Utility evaluation of product attributes: Kahneman and Tversky (16) found that consumers evaluate utility gains or losses of products or product attributes based on differences from a reference point. Wedell and Jonathan (17) also reported that for the same amount of utility gains or losses, consumers often have a higher weight on utility losses than on gains, that is, expression of loss aversion. The introduction of a decoy product may provide a reference point for consumers.

**Figure 1:**
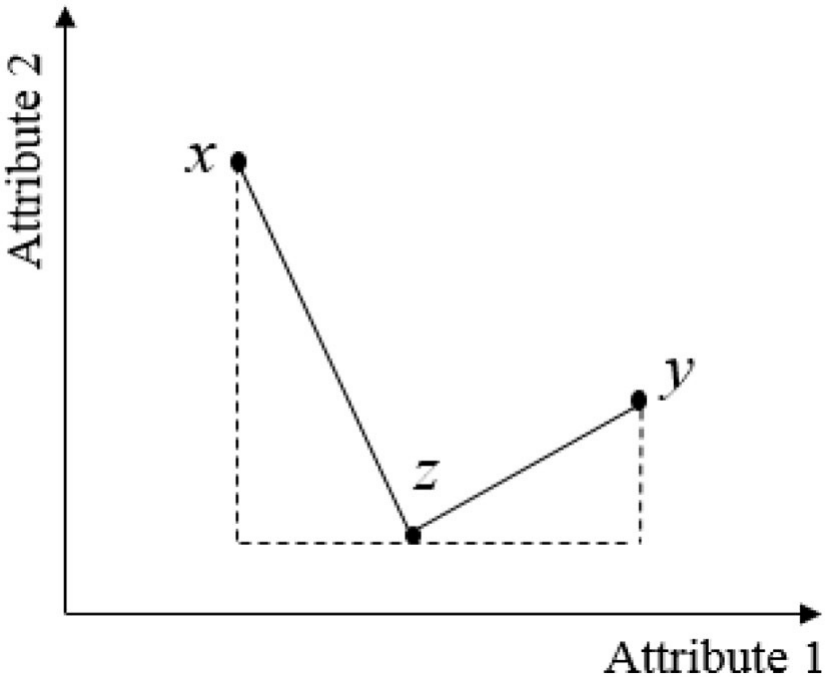
Decoy effect from the perspective of loss aversion.

As shown in Figure 1, when a decoy product *z* is added to a core set containing products *x* and *y* with *y* being the target product to change consumer preferences on, compared with the decoy product *z*, product *x* has a utility gain in attribute 1 and a loss in attribute 2, whereas the target product *y* has a utility gain in both attributes 1 and 2. Therefore, consumers may choose target product *y* to avoid the utility loss associated with product *x* in attribute 1 due to loss aversion. In this case, loss aversion affects consumer choices and can lead to a decoy effect. Ariely and Wallsten (18) suggested that the introduction of a decoy product may also change the weight consumers assign to product attributes, thereby making the target product with a higher weighted attribute more attractive. Again, as shown in Figure 1, after the decoy product *z* is introduced, if consumers give attribute 1 a higher weight, the utility gain, in other words, the attractiveness of target product *y* will increase as target product *y* is superior to product *x* in terms of attribute 1.

Chernev (19) argued that consumers give attributes that have a strong correlation with their purpose of purchase a higher weight. Müller et al. (20) also demonstrated that decoy products prompt consumer interest and that an attribute may be given a higher utility or weight by consumers if it arouses consumer interest. For example, as Chinese consumers are generally concerned about food (pork) safety, they pay greater attention to attributes reflecting pork safety information on the market, thus assign a higher utility or weight to these attributes (21). In this study, we consider quality attributes of two products: traceability and appearance, in addition to product price. Past studies have rarely considered multiple product attributes.

Research on the correlation between consumer characteristics and the decoy effect shows that consumer characteristics can affect the attribute they would like to know more about and the intensity of the decoy effect on these attributes. For example, Dhar and Glazer (22) found that consumers who have better understanding of a product are less influenced by the decoy effect. Similarly, Ratneshwar et al. (14) pointed out that consumers are prone to the decoy effect if they are unfamiliar with the product attributes. Mourali et al. (23) suggested that the decoy effect is influenced to varying degrees by consumer familiarity with the product and whether they have the intention to seek a gain or avoid a loss. Tentoria et al. (24) and Chang (25) reported that the elderly may have richer purchasing experience. However, only older consumers with expertise in the product of concern make truly rational decisions (26). Consistently, Putrevu and Lord (27) believed that experience and expertise can help consumers make decisions and that consumers more familiar with the product and more experienced in purchase and use are more rational in purchase decision-making. The aforementioned conclusions are also supported by Rao et al. (28) and Li and Zhou (29).

However, Shafir et al. (30) found that the ability of a consumer to make rational purchase decisions declines with age, and older consumers are likely to be more irrational in purchasing. Furthermore, Zhen and Yu (31) reported that consumers of all age-groups are likely to experience the decoy effect to varying degrees, except for subjects younger than 5 years as this age-group is not fully capable of identifying and evaluating products. In addition, Dholakia (32) found that the probability of irrational purchase behavior is significantly lower in men than in women. Dittmar et al. (33) confirmed that female consumers are more prone to irrational purchases, thus more likely to experience the decoy effect. Wood (34) showed that low-income consumers are also more likely to experience contextual effects. Moreover, related studies suggest that family size (35), income (36), occupation (37), marital status, and presence of minor children in a family (28) can have different degrees of impact on irrational consumer behavior.

The aforementioned studies on the correlation between consumer characteristics and the decoy effect have only investigated the relationship between one or very few individual characteristics and the decoy effect. Various individual characteristics have rarely been included in a single framework to examine the correlations between them and the decoy effect. Moreover, most existing literature in this field has focused on the purchase of general goods, with limited research conducted on food purchase. Food has the common attributes of general goods. However, given the importance of food safety to health, food has attributes that are of greater concern to consumer health. Thus, in the current study, we analyze the impact of consumer characteristics on decoy effect in food purchase. We conduct a survey of consumers in Wuxi, Jiangsu Province, China, taking traceable pork hindquarter purchase as an example. We establish the following hypotheses:

H1: There is no decoy effect in the purchase of traceable pork hindquarters.
H1-1: Assume there is a decoy effect; the effect does not change across difference product attributes.
H2: Decoy effect in the purchase of traceable pork hindquarters does not vary with demographic characteristics, which can be tested by specific hypotheses:
H2-1: Decoy effect does not vary with age.
H2-2: Decoy effect does not vary with gender.
H2-3: Decoy effect does not vary with marital status.
H2-4: Decoy effect does not vary with annual income.
H2-5: Decoy effect does not vary with family size.
H2-6: Decoy effect does not vary with the presence or absence of minors in household.
H2-7: Decoy effect does not vary with occupation.

## Survey design, implementation, and sample analysis

China is the world's largest producer and consumer of pork. China's pork production and consumption in 2018 accounted for 47.82 and 48.55% of global pork production and consumption, respectively.^1^ As pork is the most popular meat in China, consumers are very familiar with it, which allows us to avoid the possible additional decoy effect caused by unfamiliarity with the basic characteristics of the product itself (14). However, pork is also one of the food categories facing the most food safety concerns in China (38). The Chinese government has committed to developing a traceable pork market for many years. Therefore, traceable pork (specifically, traceable pork hindquarters) was selected as the target product in this study. Limiting to pork hindquarters helps reduce the need to consider the price–product dynamics of various types of pork cuts.

It should be noted that traceable pork hindquarters are not yet widely available on the Chinese market, and the various types of traceable pork hindquarters the policymakers are interested in exploring do not exist in the market. Thus, we used hypothetical pork profiles (for simplicity, traceable pork hindquarter profiles are also interchangeably referred to as traceable pork hereafter) and established the attributes of traceable pork in our design. As noted previously, consumer familiarity with a product can influence the decoy effect. As traceable pork is not yet popular on the market, we assume consumer familiarity with this product is generally identical across individuals. Therefore, using traceable pork as the target product could exclude the influence of factors other than consumer characteristics on the decoy effect.

Traceability information reflects different characteristics of different types of traceable pork. In total, three levels of traceability information were defined according to the characteristics of Chinese hog suppliers: (1) farming alone; (2) farming, slaughtering, and processing; and (3) farming, slaughtering, processing, and distribution (39). For example, if a consumer chooses pork with traceability information covering farming alone, they can only obtain information about the farming process. More traceability information makes identification of possible food safety risks more conveniently. A second pork attribute considered in this study is pork appearance. Numerous studies have shown appearance being an important factor affecting the consumer evaluation of product quality (40–43). Based on discussion with food scientists, we define pork appearance in three levels: fresh, moderate, and unappealing but palatable. Table 1 presents two types of pork products. Type 1 traceable pork contains only traceability and price information; four such products are designed and denoted by *a, b, c*, and *d*, respectively. Type 2 traceable pork considers traceability, price, and appearance; five products are created under type 2, and are denoted by e, *f* , *g, h*, and *i*, respectively.

**Table 1:** Attributes and levels of pork.

**Type 1 traceable pork**					
**Traceable pork**	***a***	***b***	***C***	***d***	
Traceable information	With traceability information on farming	With traceability information on farming, slaughtering, and wholesale	With traceability information on farming, slaughtering, and wholesale	With traceability information on farming and slaughtering	
Price (yuan/500 g; 1 yuan≈0.15 USD)	14	16	18	16	
**Type 2 traceable pork**					
**Traceable pork**	***e***	***f***	***G***	***H***	***i***
Traceable information	With traceability information on farming	With traceability information on farming, slaughtering, and wholesale	With traceability information on farming and slaughtering	With traceability information on farming and slaughtering	With traceability information on farming
Appearance	Moderate	Unappealing but palatable	Fresh	Moderate	Fresh
Price (yuan/500 g; 1 yuan≈0.15 USD)	13.5	14.5	15	15	15

Compared to non-traceable pork, the production of traceable pork with attributable information involves additional costs, which may, in turn, increase the market price of pork. As mentioned earlier, the traceable pork in this study with different traceability attributes does not currently exist on the market. Therefore, their associated prices were determined based on previous research. Specifically, as the present study was conducted in the same location (i.e., Jiangsu Province) and at a similar time as Wu et al. (44), the same price levels were chosen. Table 1 also present the prices.

Based on the aforementioned settings of traceable pork, a total of six contexts (scenarios) were designed. Contexts 1–3 were designed for type 1 traceable pork, where option *b* was the target traceable pork, and contexts 4–6 were created for type 2 traceable pork, where option *f* was the target traceable pork. The six contexts are as follows:

Context 1: With no decoy pork, survey participants were asked to choose between two subtypes of type 1 traceable pork, namely, *a* and *b* in Table 1, expressed as {*a, b*}.
Context 2: With the introduction of decoy pork *c*, participants were asked to choose among three subtypes of type 1 traceable pork, namely, *a, b*, and *c* in Table 1, expressed as {*a, b, c*}.
Context 3: With the introduction of decoy pork *d*, participants were asked to choose among three subtypes of type 1 traceable pork, namely, *a, b*, and *d* in Table 1, expressed as {*a, b, d*}.
Context 4: With no decoy pork, participants were asked to choose among three subtypes of type 2 traceable pork, namely, *e, f* , and *g* in Table 1, expressed as {*e, f, g*}.
Context 5: With the introduction of decoy pork *h*, participants were asked to choose among four subtypes of type 2 traceable pork, namely, *e, f* , *g*, and *h* in Table 1, expressed as {*e, f, g, h*}.
Context 6: With the introduction of decoy pork *i*, participants were asked to choose among four subtypes of type 2 traceable pork, namely, *e, f* , *g*, and *i* in Table 1, expressed as {*e, f, g, i*}.

H1 can be tested by calculating the purchase share of option *b* in contexts 1 and 2, with *P1 (b, a)* defined as the share of *b* in context 1, {*a, b*}, and *P2*_*c*_ *(b, a)* defined as the share of the target option *b* in context 2 after the addition of option *c*. If *P1 (b, a)* ≥ *P2*_*c*_ *(b, a)* is rejected, H1 is subsequently rejected. In other words, the “decoy effect in purchases of traceable pork” is supported [i.e., *P1 (b, a)*< *P2*_*c*_ *(b, a)*]. Similarly, H1 can also be tested by calculating the purchase share of option *b* in contexts 1 and 3, purchase share of option *f* in contexts 4 and 5, and purchase share of option *f* in contexts 4 and 6. If the null hypothesis H1 is rejected, the existence of the decoy effect is supported, that is, *P1 (b, a)*< *P3*_*d*_ *(b, a), P4 (g, e, f)*< *P5*_*h*_ *(g, e, f)*, and *P4 (g, e, f)*< *P6*_*i*_ *(g, e, f)*, respectively. Hypothesis H1-1 can be checked by testing the equality between *P2*_*c*_ *(b, a)* and *P3*_*d*_ *(b, a)* since they differ by the attribute the decoy effect is intended to operate on. Similarly, hypothesis H1-1 can be tested by examining whether *P5*_*h*_ *(g, e, f)* and *P6*_*i*_ *(g, e, f)* are equivalent. We further constructed a negative binomial count regression model based on the changes in purchases of types 1 and 2 traceable pork after the addition of decoy traceable pork *c, d, h*, and *i* in order to investigate the correlation between individual characteristics and the decoy effect, thereby testing hypotheses H2 (H2-1 to H2-7).

This study implemented a consumer survey in Wuxi, one of the first Chinese pilot cities to introduce limited traceable pork in 2010 as a joint effort by the Ministry of Commerce and the Ministry of Finance. As such, consumers in Wuxi have some basic, but not intensive, understanding of traceable pork attributes, which helps reduce consumer bias due to product unfamiliarity. Based on this, we investigated whether the decoy effect exists in the purchase of traceable pork hindquarters among consumers in Wuxi. Moreover, Wuxi is one of the largest cities in eastern China, with a high level of economic development, dense population, and wide distribution of individuals with different demographic characteristics, which contribute to the diversity and representativeness of the samples. In addition, to improve representativeness of the samples, this study was conducted in all five administrative districts of Wuxi in large- and medium-sized supermarkets, farmers' markets, and pork shops. For the sake of simplicity, 50 participants aged 18–65 years were recruited in each district, for a total of 250 participants. Every third consumer coming into view was recruited by the research team. The questionnaire was completed by local graduate students *via* face-to-face communication with the participants. The entire study was performed from 10 to 14 August 2021. In total, 241 valid questionnaires were obtained.

**Figure 2:**
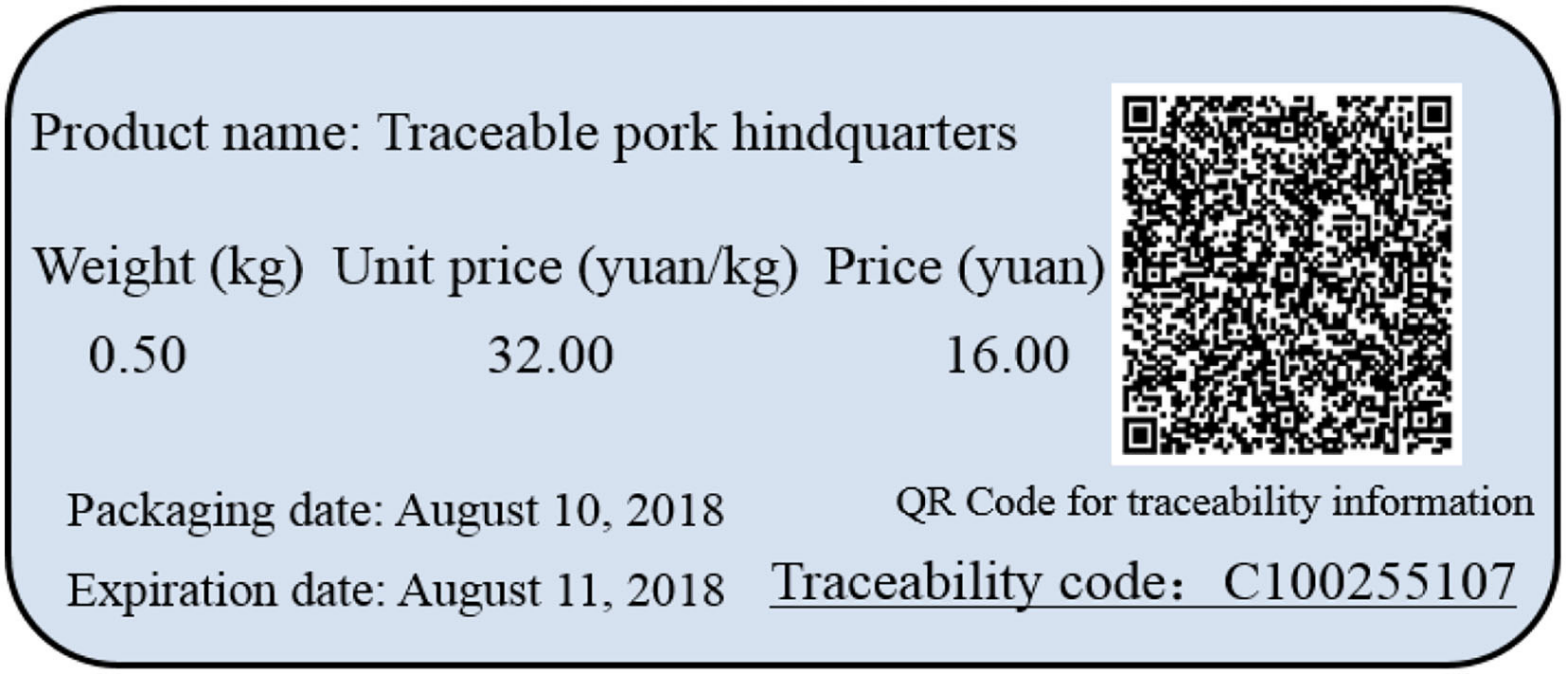
QR code for traceable pork hindquarters.

The participants were not required to make actual purchases, but actual pork products were on display at each survey site with varying levels of appearance corresponding the levels considered in this study. Each participant was asked to evaluate both types of pork, but the two types of pork were presented in random orders (either contexts 1–3 appeared first or contexts 4–6 appeared first). To resemble a real market, QR codes, as an example shown in Figure 2, were designed for each type of traceable pork. The participants could obtain information on quality and safety of the corresponding traceable pork by scanning the QR code. To remove the order effect, for type 1 traceable pork, the participants were first shown context 1, and then contexts 2 and 3 were presented in a random order. Similarly, for type 2 traceable pork, the participants were first shown context 4, and then contexts 5 and 6 were presented randomly. Each participant was paid 20 yuan (one CNY≈ 0.15 USD at the time of the study) to compensate for their time.

Participant demographic characteristics are shown in Table 2. Women accounted for 52.70% of the sample, which coincides with the fact that women are the major food shoppers in most urban families of China. In addition, 79.26% of participants were aged between 18 and 48 years, 59.75% were married, 67.64% had a junior college or college education, and 36.52% had a family size of three. The participants with an annual personal income before tax of <100,000 yuan accounted for 88.38% of the sample. Other demographics, such as the presence or absence of minor children in the family, self-reported health status, and occupation, are also listed in Table 2. It should be noted that there are certain differences between the sample and overall demographics of Wuxi. The main reason is that participants were recruited during specific hours of the day, that is, 08:00–10:00 and 16:00–18:00, two periods when most family food shopping is done. Thus, it is not surprising that the demographics of the participants randomly recruited during these time periods are not consistent with those of the urban population of Wuxi. However, this does not compromise the representativeness of the survey sample. In fact, the sample demographics of this study are generally consistent with those reported by Wu et al. (21) conducted in the same area.

**Table 2:** Participant demographics.

**Demographic**	**Category**	**Sample size (*n*)**	**Proportion (%)**
Gender	Male	114	47.30
	Female	127	52.70
Age	18–28 years	98	40.67
	29–48 years	93	38.59
	49–65 years	50	20.74
Marital status	Married	144	59.75
	Unmarried	97	40.25
Family size (*n*)	1	11	4.56
	2	39	16.18
	3	88	36.52
	4	45	18.67
	5 or more	58	24.07
Education	Primary school or below	7	2.90
	Junior high school and high school (including vocational high school)	65	26.97
	Junior college	62	25.73
	College	101	41.91
	Graduate and above	6	2.49
Personal income before tax	<50,000 yuan	135	56.02
	50,000–100,000 yuan	78	32.36
	More than 100,000 yuan	28	11.62
Presence or absence of minor children in family	Absent	139	57.68
	Present	102	42.32
Health (self-assessed)	Very poor and poor	2	0.83
	Moderate	25	10.37
	Healthy and very healthy	214	88.80
Occupation	Government employee	2	0.83
	Employee of public and private enterprises	125	51.87
	Farming	14	5.81
	Student	28	11.62
	Other	72	29.88

## Measures of the decoy effect and result

The decoy effect is measured according to Mourali et al. (23):


(1) $$\begin{array}{r} \Delta P=P_{z}\left(y;x\right)-P\left(y;x\right) \end{array}$$

where Δ*P* is the decoy effect, is the purchase share of option *y* relative to option *x* in the choice set {*x, y*}, and is the purchase share of target option *y* relative to option *x* in the choice set {*x, y, z*}, and is calculated as follows:


(2) $$\begin{array}{r} P_{z}\left(y;x\right)=\frac{P\left(y;x,z\right)}{\left[P\left(y;x,z\right)+P\left(x;y,z\right)\right]} \end{array}$$

where is the purchase share of the target option *y* relative to options *x* and *z* in the choice set {*x, y, z*} and is the purchase share of option *x* relative to options *y* and *z* in the choice set {*x, y, z*}.

**Figure 3:**
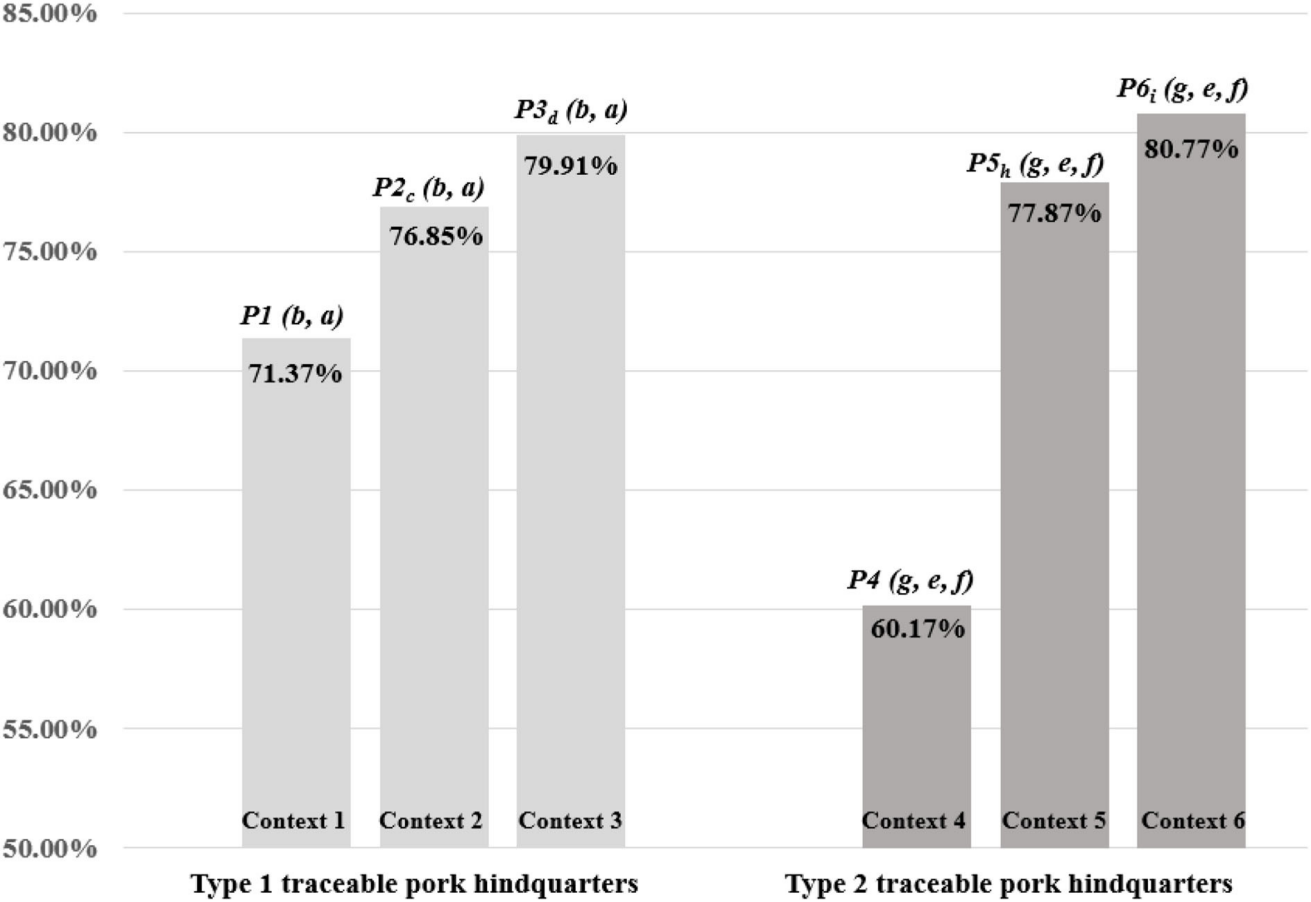
Decoy effect in types 1 and 2 traceable pork.

In context 1, that is, choice set {*a, b*}, the purchase shares of *a* and *b* were 28.63 and 71.37%, respectively. In contexts 2 and 3{*a, b, d*}, the purchase shares of target option *b* were 64.73 and 74.27%, respectively. As shown in Figure 3, the purchase share of option *b* relative to option *a* increased from 71.37% in the choice set {*a, b*} of context 1 to 76.85% in the choice set {*a, b, c*} of context 2 and to 79.91% in the choice set {*a, b, d*}of context 3, respectively. Hence, Δ*P* = 5.48% [= 42.95, *p* < 0.001] and 8.54% [χ^2^(2)= 47.11, *p* < 0.001], respectively, when comparing context 2 and 3 to context 1. Therefore, H1 is rejected, supporting *P1 (b, a)*<*P2*_*c*_ *(b, a)*, and *P1 (b, a)*<*P3*_*d*_ *(b, a)*, that is, a decoy effect exists. In contexts 4, 5, and 6, decoy effects observed after the addition of decoy traceable pork *h* and *i* on to the choice set {*e, f, g*}were Δ*P* = 17.7% [χ^2^(2) = 23.48, *p* < 0.001] and Δ*P* = 20.60% [χ^2^(2) = 31.28, *p* < 0.001], respectively. Similarly, H1 is rejected, supporting *P4 (g, e, f)*<*P5*_*h*_ *(g, e, f)*, and *P4 (g, e, f)*<*P6*_*i*_ *(g, e, f)*. Therefore, a decoy effect appears to exist in purchases of traceable pork.

As shown in Figure 3, *P3*_*d*_ *(b, a)* > *P2*_*c*_ *(b, a)* [χ^2^(2) = 25.62, *p* < 0.001] and *P6*_*i*_ *(g, e, f)* > *P5*_*h*_ *(g, e, f)* [χ^2^(2) = 13.63, *p* < 0.05]. This shows that different decoy traceable pork provided different reference points for the participants, thus inducing different levels of decoy effects. Comparing products *b* and *c*, product *c* had identical traceability information as *b* but was more expensive by two yuan. Comparing products *b* and *d*, product *d* was offered at the same price as *b* but could not reveal traceable information on the wholesale process. As a result, in this application, the decoy effect generated by a two-yuan difference is less than the traceable information on the wholesale process, providing evidence to reject H1-1. For type 2 traceable pork, both decoy products *h* and *i* had the same price as the target product *g*. Product *h* offered the same traceability information as product *g* but was less appealing in appearance (moderate vs. fresh). Product *i* was at the same level of appearance as target product *g* but did not offer traceability information regarding the slaughtering process. The result indicated that product *i* generated a stronger decoy effect than product *h*, thus also rejecting H1-1.

## Regression analysis

We further adopted a negative binomial count regression model to investigate the correlations between individual characteristics and the decoy effect, thereby testing hypotheses H2 and H2_1_ to H2_7_. In the current study, under the decoy effect, the participants changed their purchase decision due to the presence of decoy products. We define *y*_*i*_ as the number of times consumers changed their decision from a competitive traceable pork to the target traceable pork after the addition of decoy traceable pork *c, d, h*, and *i*. Therefore, *y*_*i*_ can take a value of 0, 1, 2, 3, or 4. As the dependent variable is a non-negative integer and to allow over-dispersion, we used a negative binomial count regression model with a probability of *y*_*i*_ defined as follows:


(3) $$\begin{array}{r} P\left\{yi\right\}=\frac{\lambda^{y_{i}}}{y_{i}!}e^{-\lambda }\;y_{i}=0,1,2,3,4 \end{array}$$

where λ is a parameter taking only positive values. In addition, it is assumed that parameter λ is determined by dependent variables *X*_*i*_. The negative binomial count model can then be estimated by maximum simulated likelihood over sample N:


(4) $$\begin{array}{r} \overset{N}{\underset{i=1}{\sum }}Ln\left(\frac{\text{1}}{K}\overset{K}{\underset{j=1}{\sum }}\tilde{f}\left(y_{i}|X_{i},\theta ,{u^{j}}_{i}\right)\right) \end{array}$$

where θ> 0, μ_*i*_= $e^{{X_{i}}^{\prime }\beta }$, and β > 0. θ is a shape parameter, β is a scale parameter, K is the number of simulations, and *X*_*i*_ is a group of demographics affecting the decoy effect. Table 3 presents the definition and measurement of each variable. The estimation was performed using Stata 14.0, and the results are shown in Table 4.

**Table 3:** Definition and measurement of variables.

**Variable**	**Definition**	**Mean**
18–25 years	“18–25 years” was used as reference group	
29–48 years (X_1_)	Dummy variable. Yes = 1; No = 0	0.39
49–65 years (X_2_)	Dummy variable. Yes = 1; No = 0	0.21
Male (X_3_)	Dummy variable. Yes = 1; No = 0	0.47
Married (X_4_)	Dummy variable. Yes = 1; No = 0	0.60
Annual personal income <50,000 yuan (all pre-tax)	“Annual personal income <50,000 yuan”	
	was used as the reference group	
Annual personal income between 50,000 and 100,000 yuan (X_5_)	Dummy variable. Yes = 1; No = 0	0.32
Annual personal income more than 100,000 yuan (X_6_)	Dummy variable. Yes = 1; No = 0	0.12
Family size of 1 or 2	“Family size of 1 or 2” was used as the reference group	
Family size of 3 or 4 (X_7_)	Dummy variable. Yes = 1; No = 0	0.55
Family size of 5 or more (X_8_)	Dummy variable. Yes = 1; No = 0	0.24
Presence of minor children in household (X_9_)	Dummy variable. Yes = 1; No = 0	0.42
Other occupations	“Other occupations” was used as the reference group	
Government employee (X_10_)	Dummy variable. Yes = 1; No = 0	0.01
Employee of enterprises (X_11_)	Dummy variable. Yes = 1; No = 0	0.52
Farmer (X_12_)	Dummy variable. Yes = 1; No = 0	0.06
Student (X_13_)	Dummy variable. Yes = 1; No = 0	0.12

**Table 4:** Negative binomial count regression model estimation result.

**Variable**	**Coef.**	**Sta. Err.**	***Z***	***P***
29–48 years (X_1_)	0.028	0.168	0.16	0.870
49–65 years (X_2_)	0.365	0.188	1.94	0.052
Male (X_3_)	0.057	0.109	0.52	0.604
Married (X_4_)	−0.396^**^	0.143	−2.77	0.006
Annual personal income between 50,000 and 100,000 yuan (X_5_)	−0.510^**^	0.148	−3.44	0.001
Annual personal income more than 100,000 yuan (X_6_)	−0.653^**^	0.247	−2.65	0.008
Family size of 3 or 4 (X_7_)	−0.028	0.144	−0.19	0.847
Family size of 5 or more (X_8_)	−0.175	0.165	−1.06	0.291
Presence of minor children in household (X_9_)	−0.423^**^	0.125	−3.38	0.001
Government employee (X_10_)	0.531	0.532	1.00	0.318
Employee of enterprises (X_11_)	0.081	0.145	0.56	0.575
Farmer (X_12_)	0.396^*^	0.199	1.99	0.047
Student (X_13_)	−0.048	0.210	−0.23	0.817
Constant	0.862	0.221	3.90	0.000

** Significance at the 1% level.

* Significance at the 5% level.

LR chi2 (13) = 63.610; Prob>chi2 = 0.000; Pseudo R^2^ = 0.080; log likelihood = −364.517.

As shown in Table 4, variable X_1_ (29- to 48-year age-group) and variable X_2_ (49- to 65-year age-group) were not significant; thus, H2-1 could not be rejected. Variable X_3_ (male participant) was also not significant; thus, H2-2 could not be rejected. The coefficient of the variable representing whether the participant was married (X_4_) was negative and significant at the 1% level; thus, H2-3 could be rejected. Compared with unmarried participants, married participants were less likely to experience the decoy effect.

The coefficients of variables X_5_ and X_6_ (representing the annual income of 50,000–100,000 yuan and more than 100,000 yuan, respectively) were negative and significant at the 1% level, thus rejecting H2-4. Therefore, compared with participants with an annual pre-tax income of <50,000 yuan, those with a higher annual income were less likely to experience the decoy effect. This result is consistent with the conclusions of Wood (34) but differs from that of Lin and Lin (36). Variables X_7_ (family size of 3 or 4) and X_8_ (family size of 5 or more) were not significant, so H2-5 could not be rejected. The coefficient of the variable X_9_ (presence of minor children in the household) was negative and significant at the 1% level; thus, H2-6 could be rejected. This indicated that compared with participants who did not have minor children at home, those who did were less likely to experience the decoy effect. This differs from the conclusions of Rao et al. (28). Variables X_10_ (government employee), X_11_ (employee of an enterprise), and X_13_ (student) were all insignificant. However, the coefficient of variable X_12_ (farmer) was positive and significant at the 5% level. This indicates that compared with other types of occupation, farmers were more likely to experience the decoy effect. Thus, H2-7 could be rejected. Table 5 reports the marginal effects. When calculating the marginal effect of a single dummy variable, all other variables were measured at the sample median.

**Table 5:** Marginal effect of individual characteristic variables.

**Variable**	**Marginal effect**	**Sta. Err.**	***Z***	***P***
29–48 years (X_1_)	0.045	0.260	0.17	0.862
49–65 years (X_2_)	0.564	0.294	1.92	0.055
Male (X_3_)	0.087	0.168	0.52	0.604
Married (X_4_)	−0.602^**^	0.224	−2.69	0.007
Annual personal income between 50,000 and 100,000 yuan (X_5_)	−0.164^**^	0.232	−3.32	0.001
Annual personal income more than 100,000 yuan (X_6_)	−0.974^*^	0.385	−2.53	0.011
Family size of 3 or 4 (X_7_)	−0.041	0.222	−0.18	0.853
Family size of 5 or more (X_8_)	−0.264	0.255	−1.04	0.300
Presence of minor children in household (X_9_)	−0.645^**^	0.196	−3.28	0.001
Government employee (X_10_)	0.798	0.824	0.97	0.333
Employee of enterprises (X_11_)	0.128	0.224	0.57	0.568
Farmer (X_12_)	0.602	0.308	1.95	0.051
Student (X_13_)	−0.069	0.325	−0.21	0.833

* Significance at the 5% level.

** Significance at the 1% level.

Based on Table 5, the marginal effect of variable X_4_ (whether the participant was married) was negative (−0.602) and significant at the 1% level. This suggests that married participants made 0.602 less changes in their product choice due to the decoy effect than unmarried participants. As the total possible number of changes was 4, the reduction in the number of changes among married participants was 15.05% relative to those participants who were unmarried. The marginal effects of variables X_5_ (annual income between 50,000 and 100,000 yuan) and X_6_ (more than 100,000 yuan) were negative (−0.164 and −0.974, respectively) and significant at the 1 and 5% levels, respectively. Specifically, compared with participants with an annual income of <50,000 yuan, the number of changes in purchase decision due to decoy traceable pork was reduced by 0.164 among participants with an income of 50,000–100,000 yuan, or 4%, and by 0.974 among participants with an annual income of more than 100,000 yuan, or 24.35%. Finally, the marginal effect of variable X_9_ (minor children in the family) was also negative (−0.645) and significant at the 1% level. Compared with participants without minor children at home, the number of changes in purchase decision due to decoy traceable pork was reduced by 0.645 among participants with minor children. This represented a reduction of 16.12%.

## Conclusion and implications

This study investigated whether the decoy effect may exist in the purchases of food, whether the effect may differ across product attributes, and whether there is correlation between the decoy effect and individual consumer characteristics. Based on an in-person consumer survey in Wuxi, China, on traceable pork hindquarters, similar to other types of consumer products, we identified decoy effects in all scenarios we considered. Moreover, we show evidence that the decoy effect varied with product attributes, and consumer individual characteristics have strong correlation with how they make product choices given decoy products.

This study can be useful for more accurately assessing patterns of consumer food purchases, product marketing, and developing traceable food markets in China. Consumers are the major actors of traceable food market, and the effective establishment of China traceable food market inherently depends on consumer purchasing behavior. For consumer behavior and marketing, since there is no formalized food traceability system in China, traceable food tends to be marketed with different levels of traceability information. Together with other types of food attributes, this creates room for the decoy effect to influence consumer choices. Our study shows that different consumers react differently to decoy effects on different types of product attributes. With proper consideration of the decoy effect and better knowledge on the consumer profile, marketers will be able to better measure consumer choices and make more precise predictions of the market, particularly when new (traceable) products are introduced to the market. For policymakers, if traceability is deemed to be useful to consumers, after careful cost and benefit assessment, public education and information programs could take advantage of the decoy effect to nudge consumers to make choices supporting a formal and systematic scheme of food traceability. However, the use of the effect to promote the development of China traceable food market discussed here is an auxiliary strategy and is by no means a long-term solution. The key to the development of China traceable food market is that the government should be committed to developing a national unified traceable food standard system, reducing the cost of producers adopting traceable food production standards, and maintaining the stability of traceable food prices to increase the consumption.

This study has some limitations. First, the analysis is based on data from a single city in China. Due to large regional differences in China and between countries, applicability and generalizability of our results should be verified on a larger scale. Second, despite that the intention of this study is not to obtain an unbiased estimate of consumer willingness to pay for traceable pork *per se*, we hope to offer reliable analysis and add to the related literature. Although we have displayed actual pork products during our survey, consumers made decisions hypothetically and did not involve actual payment. Consumers may have misrepresented their actual purchase intention (41, 45). The discrepancy between hypothetical and actual behavior is known as hypothetical bias, and a number of approaches have been proposed in the literature to reduce such bias (46, 47). A future study like ours can take advantage of these mitigation methods. Furthermore, revealed preference methods such as experimental auction may be used to investigate the decoy effect in food purchases. In particular, this study confirmed that the decoy effect varies according to the attributes of pork hindquarters and that the individual characteristics of consumers are closely related to consumer choices based on decoy pork. However, as the decoy effect is an irrational behavior of consumers, this study does not encourage the abuse of the decoy effect in food purchase, especially the use of the decoy effect to market foods that do not comply with laws and regulations. This is the greatest drawback of this study. Overall, this study aims to promote the construction of traceable food systems in China through marketing strategies employing the decoy effect based on the Chinese situation so that consumers pay more attention to and use traceability information, thereby presenting an auxiliary strategy for promoting traceable food systems in China.

## Data availability statement

The raw data supporting the conclusions of this article will be made available by the authors, without undue reservation.

## Ethics statement

The studies involving human participants were reviewed and approved by Jiangnan University. Written informed consent for participation was not required for this study in accordance with the national legislation and the institutional requirements.

## Author contributions

All authors listed have made a substantial, direct, and intellectual contribution to the work and approved it for publication.

## Funding

This study was financially supported by the National Social Science Fund of China: Research on Social Co-governance of Food Safety Risks and Cross-border Cooperative Governance Mechanisms (20&ZD117).

## Conflict of interest

The authors declare that the research was conducted in the absence of any commercial or financial relationships that could be construed as a potential conflict of interest.

## Publisher's note

All claims expressed in this article are solely those of the authors and do not necessarily represent those of their affiliated organizations, or those of the publisher, the editors and the reviewers. Any product that may be evaluated in this article, or claim that may be made by its manufacturer, is not guaranteed or endorsed by the publisher.

## Footnotes

^1^Data source: National Bureau of Statistics of China (ed.): China Statistical Yearbook 2018, China Statistics Press, 2018.

## References

[1] Gintis H. The Bounds of Reason: Game Theory and the Unification of Bahavion Scineces. New Jersey, NJ: Princeton University Press (2009). p. 116.

[2] Heath TB Chatterjee S. Asymmetric decoy effects on lower-quality versus higher-quality brands: meta-analytic and experimental evidence. J Consumer Res. (1995) 3:268–84. 10.1086/209449

[3] Huber J Payne JW Puto C. Adding asymmetrically dominated alternatives: violations of regularity and the similarity hypothesis. J Consumer Res. (1982) 9:90–8. 10.1086/208899

[4] Prelec D Birger W Florian Z. The role of inference in context effects: inferring what you want from what is available. J Consumer Res. (1997) 24:118–25. 10.1086/209498

[5] Gonzalez PD Sallan JM Simo P Carrion R. Effects of the addition of simple and double decoys on the purchasing process of airline tickets. J Air Transport Manag. (2013) 29:39–45. 10.1016/j.jairtraman.2013.02.002

[6] Lin C-H Sun Y-C Chuang S-C Su HJ. Time pressure and the compromise and attraction effects in choice. In: Lee AY, Soman D, editors. Advances in Consumer Research, Vol. 35. Duluth, MN: Association for Consumer Research (2008). p. 348–52.

[7] Malkoc SA Hoeffler S Hedgcock W. Valence asymmetries in preference: the case of attraction effect. Adv Consum Res. (2008) 35:123–32. Available online at: https://www.researchgate.net/publication/256010704_Valence_Asymmetries_in_Preference_The_Case_of_Attraction_Effect

[8] Malkoc SA Hedgcock W Hoeffler S. The failure of the attraction effect among unattractive alternatives. J Consumer Psychol. (2013) 23:317–29. 10.1016/j.jcps.2012.10.008

[9] Frederick S Leonard L Ernest B. The limits of attraction. J Market Res. (2014) 51:487–507. 10.1509/jmr.12.0061

[10] Lichters M Bengart P Sarstedt M Vogt B. What really matters in attraction effect research: when choices have economic consequences. Mark Lett. (2015) 28:127–38. 10.1007/s11002-015-9394-6

[11] Wu LH Liu PP Chen XJ Hu WY Chen YH. Decoy effect in food appearance, traceability, and price: case of consumer preference for pork hindquarter. J Behav Exp Econ. (2020) 87:101553. 10.1016/j.socec.2020.101553

[12] Dhar R Maach MB. Toward extending the compromise effect to complex buying contexts. J Market Res. (2004) 41:258–61. 10.1509/jmkr.41.3.258.35996

[13] Tversky A Kahneman D. Judgment under uncertainty: heuristics and biases. Science. (1974) 185:1124–31. 10.1126/science.185.4157.1124 17835457

[14] Ratneshwar S Shocker AD Stewart DW. Toward understanding the attraction effect: the implications of product stimulus meaningfulness and familiarity. J Consumer Res. (1987) 13:520–33. 10.1086/209085

[15] Brenner L Rottenstreich Y. Comparison, grouping, and preference. Psychol Sci. (1999) 10:225–9. 10.1111/1467-9280.00141

[16] Kahneman D Tversky A. Prospect theory: an analysis of decision under risk. Econometrica. (1979) 47:263–91. 10.2307/1914185

[17] Wedell D Jonathan P. Using judgments to understand decoy effects in choice. Org Behav Hum Decision Proces. (1996) 67:326–44. 10.1006/obhd.1996.0083

[18] Ariely D Wallsten T. Seeking subjective dominance in multidimensional space: an explanation of the asymmetric dominance effect. Org Behav Hum Decision Proces. (1995) 63:223–32. 10.1006/obhd.1995.1075

[19] Chernev A. Feature complementarity and assortment in choice. J Consumer Res. (2005) 31:748–59. 10.1086/426608

[20] Müller H Victor S Sebastian L. Prize decoys at work — new experimental evidence for asymmetric dominance effects in choices on prizes in competitions. Int J Res Market. (2014) 31:457–60. 10.1016/j.ijresmar.2014.09.003

[21] Wu LH Wang HS Zhu D Hu WY Wang SX. Chinese consumers' willingness to pay for pork traceability information-the case of Wuxi. Agri Econ. (2016) 47:71–9. 10.1111/agec.12210

[22] Dhar R Glazer R. Similarity in context: cognitive representation and violation of preference and perceptual invariance in consumer choice. Org Behav Hum Decision Proces. (2007) 67:280–93. 10.1006/obhd.1996.0080

[23] Mourali M Böckenholt U Laroche M. Compromise and attraction effects under prevention and promotion motivations. J Consumer Res. (2007) 34:234–47. 10.1086/519151

[24] Tentori K Daniel O Lynn H Cynthia M. Wisdom and aging: irrational preferences in college students but not older adults. Cognition. (2001) 81:B87–96. 10.1016/S0010-0277(01)00137-8 11483173

[25] Chang YW. The impacts of domain knowledge and personal traits on decoy effects. J Manage. (2016) 10:36–57.

[26] Kim S Hasher L. The attraction effect in decision making: superior performance by older adults. Quart J Exp Psychol. (2005) 58:120–33. 10.1080/02724980443000160 15881294 PMC1751469

[27] Putrevu S Lord K. Search dimensions, patterns and segment profiles of grocery shoppers. J Retail Consumer Serv. (2001) 8:127–37. 10.1016/S0969-6989(00)00013-8

[28] Rao W Min ZT Wei XJ. The relationship between irrational decision-making and demographics of high-tech venture entrepreneurs. Statisti Decision. (2011) 19:87–9. 10.13546/j.cnki.tjyjc.2011.19.056

[29] Li S Zhou TR. Comparative analysis of consumers' rational and irrational value preference. Price Theory Practic. (2011) 3:70–1. 10.19851/j.cnki.cn11-1010/f.2011.03.034

[30] Shafir S Tom W Brian S. Context-dependent violations of rational choice in honeybees (apis mellifera) and gray jays (perisoreus canadensis). Behav Ecol Sociobiol. (2002) 51:180–7. 10.1007/s00265-001-0420-8

[31] Zhen SS Yu RJ. The development of the asymmetrically dominated decoy effect in young children. Sci Rep. (2016) 6:22678. 10.1038/srep22678 26935899 PMC4776153

[32] Dholakia U. Temptation and resistance: an integrated model of consumption impulse formation and enactment. Psychol Market. (2000) 17:955–82. 10.1002/1520-6793(200011)17:11<955::AID-MAR3>3.0.CO;2-J

[33] Dittmar H Jane B Susanne F. Gender identity and material symbols: objects and decision considerations in impulse purchases. J Econ Psychol. (1995) 16:480–91. 10.1016/0167-4870(95)00023-H8826795

[34] Wood M. Socio-economic status, delay of gratification, and impulse buying. J Econ Psychol. (1998) 19:295–320. 10.1016/S0167-4870(98)00009-9

[35] Liu Y. Allocation of Family Financial Assets. (Dissertation), Southwestern University of Finance and Economics, Chengdu, China (2005).

[36] Lin CH Lin HM. An exploration of Taiwanese adolescents' impulsive buying tendency. Adolescence. (2005) 40:215–23. 15861627

[37] Kühberger A. The influence of framing on risky decisions: a meta-analysis. Organ Behav Hum Decis Process. (1998) 75:23–55. 10.1006/obhd.1998.2781 9719656

[38] Yin SJ Li R Wu LH Chen XJ. China Food Safety Development Report. Beijing: Peking University Press (2018). p. 318.

[39] Wu LH Wang SX Zhu D. Research on consumer food traceability attribute preference: based on choice based conjoint analysis. J Agrotechn Econ. (2015) 4:45–53. 10.13246/j.cnki.jae.2015.04.006

[40] Grunert K. What is in a steak? A cross-cultural study on the quality perception of beef. Food Qual Pref. (1997) 8:157–74. 10.1016/S0950-3293(96)00038-9

[41] Roosen J Jayson L John F. Consumer demand for and attitudes toward alternative beef labeling strategies in France, Germany, and the UK. Agribusiness. (2003) 19:77–90. 10.1002/agr.10041

[42] Alfnes F Guttormsen AG Steine G Kolstad K. Consumers' willingness to pay for the color of salmon: a choice experiment with real economic incentives. Am J Agric Econ. (2006) 88:1050–61. 10.1111/j.1467-8276.2006.00915.x

[43] Meenakshi JV Banerji A Manyong V Tomlins K Mittal N Hamukwala P. Using a discrete choice experiment to elicit the demand for a nutritious food: willingness-to-pay for orange maize in rural Zambia. J Health Econ. (2012) 31:62–71. 10.1016/j.jhealeco.2012.01.002 22317960

[44] Wu LH Gong XR Chen XJ Zhu D. Consumption preferences for traceable information attributes with ex ante quality assurance and ex post traceability. China Popul Resour Environ. (2018) 28:148–60. 10.12062/cpre.2018042231878189

[45] Wu LH Qin SS Zhu D Li QG Hu WY. Consumer preferences for origin and traceability information of traceable pork. Chinese Rural Econ. (2015) 6:47–62.34276494

[46] Penn J Hu WY. Understanding hypothetical bias: an enhanced meta-analysis. Am J Agric Econ. (2018) 100:1186–206. 10.1093/ajae/aay021

[47] Penn J Hu WY. Cheap talk efficacy under potential and actual hypothetical bias: a meta-analysis. J Environ Econ Manage. (2019) 96:22–35. 10.1016/j.jeem.2019.02.005

